# Management and treatment of children, young people and adults with systemic lupus erythematosus: British Society for Rheumatology guideline scope

**DOI:** 10.1093/rap/rkad093

**Published:** 2023-12-05

**Authors:** Md Yuzaiful Md Yusof, Eve M D Smith, Sammy Ainsworth, Kate Armon, Michael W Beresford, Morgan Brown, Lindsey Cherry, Christopher J Edwards, Kalveer Flora, Rebecca Gilman, Bridget Griffiths, Caroline Gordon, Paul Howard, David Isenberg, Natasha Jordan, Arvind Kaul, Peter Lanyon, Philip M Laws, Liz Lightsone, Hanna Lythgoe, Christian D Mallen, Stephen D Marks, Naomi Maxwell, Elena Moraitis, Clare Nash, Ruth J Pepper, Clarissa Pilkington, Antonios Psarras, Heather Rostron, Jade Skeates, Sarah Skeoch, Dalila Tremarias, Chris Wincup, Asad Zoma, Edward M Vital

**Affiliations:** Leeds Institute of Rheumatic and Musculoskeletal Medicine, University of Leeds, Chapel Allerton Hospital, Leeds, UK; NIHR Leeds Biomedical Research Centre, Leeds Teaching Hospitals NHS Trust, Leeds, UK; Department of Women’s and Children's Health, Institute of Life Course and Medical Sciences, University of Liverpool Faculty of Health and Life Sciences, Liverpool, UK; Department of Paediatric Rheumatology, Alder Hey Children’s NHS Foundation Trust, Liverpool, UK; LUPUS UK, Romford, UK; Department of Paediatrics, Cambridge University Hospitals NHS Foundation Trust, Cambridge, UK; Department of Women’s and Children's Health, Institute of Life Course and Medical Sciences, University of Liverpool Faculty of Health and Life Sciences, Liverpool, UK; Department of Paediatric Rheumatology, Alder Hey Children’s NHS Foundation Trust, Liverpool, UK; LUPUS UK, Romford, UK; School of Health Sciences, University of Southampton, Southampton, UK; Musculoskeletal Research Unit, NIHR Southampton Clinical Research Facility, University Hospital Southampton, Southampton, UK; Department of Rheumatology, Northwick Park Hospital, London North West University Healthcare NHS Trust, Harrow, UK; Rheumatology Research Group, Institute of Inflammation and Ageing, College of Medical and Dental Sciences, University of Birmingham, Birmingham, UK; Department of Rheumatology, Freeman Hospital, Newcastle, UK; Rheumatology Research Group, Institute of Inflammation and Ageing, College of Medical and Dental Sciences, University of Birmingham, Birmingham, UK; LUPUS UK, Romford, UK; Centre for Rheumatology, Division of Medicine, University College London, London, UK; Department of Adolescent Rheumatology, St James’s Hospital and Children’s Health Ireland, Dublin, Ireland; Department of Rheumatology, St George’s University Hospitals NHS Foundation Trust, London, UK; Lifespan and Population Health, School of Medicine, University of Nottingham, Nottingham, UK; Nottingham University Hospitals NHS Trust, Nottingham, UK; Department of Dermatology, Leeds Teaching Hospitals NHS Trust, Leeds, UK; Centre for Inflammatory Disease, Department of Immunology and Inflammation, Imperial College London, London, UK; Department of Paediatric Rheumatology, Manchester University NHS Foundation Trust, Manchester, UK; Centre for Musculoskeletal Health Research, School of Medicine, Keele University, Keele, UK; Department of Paediatric Nephrology, Great Ormond Street Hospital for Children NHS Foundation Trust, London, UK; NIHR Great Ormond Street Hospital Biomedical Research Centre, University College London Great Ormond Street Institute of Child Health, London, UK; LUPUS UK, Romford, UK; Department of Paediatric Rheumatology, Great Ormond Street Hospital NHS Foundation Trust, London, UK; Infection, Immunity and Inflammation Department, University College of London Great Ormond Street Institute of Child Health, London, UK; Pharmacy Department, Sheffield Children’s NHS Foundation Trust, Sheffield, UK; Department of Renal Medicine, Royal Free Hospital, London, UK; Department of Paediatric Rheumatology, Great Ormond Street Hospital NHS Foundation Trust, London, UK; Kennedy Institute of Rheumatology, Nuffield Department of Orthopaedics, Rheumatology and Musculoskeletal Sciences, University of Oxford, Oxford, UK; Leeds Institute of Rheumatic and Musculoskeletal Medicine, University of Leeds, Chapel Allerton Hospital, Leeds, UK; Leeds Children’s Hospital, Leeds Teaching Hospitals NHS Trust, Leeds, UK; Royal National Hospital for Rheumatic Diseases, Royal United Hospitals Bath NHS Foundation Trust, Bath, UK; Royal National Hospital for Rheumatic Diseases, Royal United Hospitals Bath NHS Foundation Trust, Bath, UK; LUPUS UK, Romford, UK; Department of Clinical and Academic Rheumatology, King’s College Hospital NHS Foundation Trust, London, UK; Lanarkshire Centre for Rheumatology, Hairmyres Hospital, East Kilbride, Scotland, UK; Leeds Institute of Rheumatic and Musculoskeletal Medicine, University of Leeds, Chapel Allerton Hospital, Leeds, UK; NIHR Leeds Biomedical Research Centre, Leeds Teaching Hospitals NHS Trust, Leeds, UK

**Keywords:** biologic DMARDs, guideline, lupus nephritis, management, systemic lupus erythematosus

## Abstract

The objective of this guideline is to provide up-to-date, evidence-based recommendations for the management of SLE that builds upon the existing treatment guideline for adults living with SLE published in 2017. This will incorporate advances in the assessment, diagnosis, monitoring, non-pharmacological and pharmacological management of SLE. General approaches to management as well as organ-specific treatment, including lupus nephritis and cutaneous lupus, will be covered. This will be the first guideline in SLE using a whole life course approach from childhood through adolescence and adulthood. The guideline will be developed with people with SLE as an important target audience in addition to healthcare professionals. It will include guidance related to emerging approved therapies and account for National Institute for Health and Care Excellence Technology Appraisals, National Health Service England clinical commissioning policies and national guidance relevant to SLE. The guideline will be developed using the methods and rigorous processes outlined in ‘Creating Clinical Guidelines: Our Protocol’ by the British Society for Rheumatology.


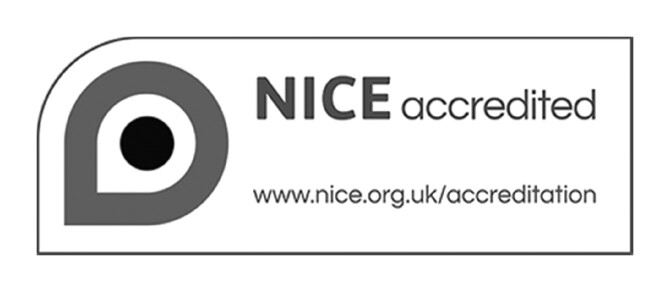


NICE has accredited the process used by BSR to create its clinical guidelines. The term began on 27 February 2012 and the current renewed accreditation is valid until 31 December 2023. More information on accreditation can be viewed at www.nice.org.uk/accreditation.

## Introduction

### Why is the guideline needed?

The 2017 BSR guideline for the management of adults with SLE was the first UK-based clinical guideline for this condition. It was comprehensive and included various aspects of diagnosis, assessment, monitoring and treatment of mild, moderate and severe active lupus in adults with SLE [1]. This guideline was an important step forward to harmonize the investigations and treatment of adults living with SLE across the UK. The literature search underpinning that guideline included manuscripts published until June 2015. Since the development and publication of that guideline there have been rapid advances in the diagnosis, assessment and therapeutic management of SLE warranting an update to that guideline. There is a need for a consistent approach to the diagnosis and treatment of SLE across the UK to address various issues, including inconsistency in diagnostics (e.g. requirement for a positive ANA and use of classification criteria) and access to treatment and how these result in inequity for people with SLE. A revised, evidence-based guideline can address these issues. This update will use the National Institute for Health and Care Excellence (NICE) accreditation process that applies to all BSR guidelines.

In the existing BSR guideline, for adults with moderate–severe non-renal SLE, the only licensed biologic therapy at the time of the literature search was belimumab (B cell activating factor inhibitor), while rituximab (anti-CD20 monoclonal antibody) was commissioned through the NHS England (NHSE) policy. Since then, new therapies have been licenced for adult SLE, such as belimumab in LN, voclosporin (a calcineurin inhibitor) in LN and anifrolumab (a type I IFN receptor monoclonal antibody) in non-renal SLE. However, for the more recently licensed treatments, licensing does not currently extend to the paediatric age group. Each of these therapies comes with nuances regarding those who are most likely to benefit. Guidance is now needed on how these new therapies fit within UK practice that already includes many off-label therapies.

The most significant developments in evidence are for LN. The existing SLE guideline endorsed the LN guidance from the 2012 EULAR and European Renal Association–European Dialysis and Transplant Association (ERA-EDTA) recommendations for the management of LN [2]. Since that original publication, the historic standard of care (mycophenolate with corticosteroid) is now known to be inferior to several subsequently licensed combinations as well as other off-label combinations for remission induction of LN [3, 4]. An aggressive multitargeted approach from the outset has been suggested to improve short- and long-term outcomes of people with LN [2, 5].

The 2017 BSR guideline did not include children and young people. European recommendations for the diagnosis and management of lupus and LN in children and young people were developed as part of the Single Hub and Access Point for Paediatric Rheumatology in Europe (SHARE) initiative and published in 2017 [6, 7]. These recommendations have provided an important basis for evidence-informed, consensus-led guidance to paediatricians but, similar to adult-onset SLE, additional evidence has subsequently emerged and updated guidelines are also warranted [8]. A whole life course approach guideline by BSR for other conditions, e.g. inflammatory myopathy, has been successfully completed [9]. Therefore, in order to promote equity in care and treatment, we feel it is important to represent the needs of children and young people in whom evidence from adults may have relevance.

### Key facts and figures

SLE is a lifelong autoimmune disease that causes inflammation and subsequent damage to a range of organs, including skin, joints, kidneys, brain, lungs and heart. The exact cause of SLE remains unknown, although combinations of genetic, environmental, immunological and hormonal factors are thought to play a key role. The course of SLE is typically characterized by periods of relapse and remission following treatments. It can present at any age, with 15–20% of people developing it in childhood. Among adults, females are nine times more likely to be affected than males, with a peak incidence in the reproductive years [10]. This gender difference is less marked in childhood, especially pre-puberty [11]. SLE has been estimated to affect ≈1 in 1000 adults in the UK [12]. It is most frequently observed in people of African-Caribbean and South Asian ancestry, so prevalence will vary in line with regional demographics [12, 13]. In these ancestral groups, the disease tends to be more severe compared with people of European ancestry, with a higher incidence of renal involvement in particular [14]. Juvenile-onset SLE (JSLE) tends to have a more aggressive presentation and disease course compared with adult-onset SLE [15]. In particular, there are higher numbers of children who have renal and neurological complications [16], while 20% of children accrue organ damage within 1 year of diagnosis [17].

Although SLE and JSLE are uncommon, they have substantial unmet needs since mortality is increased by ≈18-fold in JSLE and 3-fold in adult-onset SLE compared with the general population, particularly if LN occurs, and much higher in those with LN than without [15, 18–20]. Death from active SLE is rare in the UK, with the top three causes of mortality being attributed to infection, cancer and cardiovascular disease [21]. In a UK lupus cohort study in which people with SLE were followed for up to 21 years, a 10% mortality was recently reported, with the mean age of death being 53.7 years [22]. In another UK lupus cohort study with a 40-year follow-up that compared survival rates in people diagnosed between 2006 and 2011 with those diagnosed between 1978 and 1989, a significant improvement in survival was seen over the last 40 years [23]. Despite this, adults with SLE still die on average 25 years earlier than expected [22], with mortality rates of up to 10% within 10 years of JSLE diagnosis [24].

A key determinant of short- and long-term outcomes for SLE is early diagnosis and intervention, with the first year after diagnosis being a particularly high-risk time, associated with the highest standardized mortality ratio [25]. A survey by Lupus UK has demonstrated the delay from symptom onset to diagnosis across the UK to be up to 6.4 years [26]. This is particularly concerning as it is known that even a 6-month delay in diagnosis has been associated with greater healthcare utilization and flares [27]. SLE costs the UK economy ≈£8000 per person per year in direct and indirect healthcare costs. These costs remain high after diagnosis and increase with increasing disease severity [28, 29]. Furthermore, SLE negatively affects patients’ daily lives, work productivity and career choices [30] and significantly impairs quality of life, similar to other chronic diseases such as congestive cardiac failure but at a younger age [31].

### Current practice

People with suspected lupus are referred to a physician and multidisciplinary team with experience in managing the disease in an appropriate and timely manner. They confirm the diagnosis by using clinical grounds and the presence of characteristic immunological abnormalities, although other investigations and follow-up may be needed. Specialists can assess the level of disease activity and provide advice on treatment and monitoring of the disease, its complications and potential side effects of therapy. Current approaches to management are generally centred on rheumatology clinics in a secondary or tertiary care setting with appropriate multidisciplinary team involvement, including transition to adolescent and adult services and cross-referral to other relevant specialties such as dermatology, nephrology, immunology, haematology, obstetrics/maternal medicine physicians, respiratory medicine, neurology, cardiology, gastroenterology, neuropsychiatry, psychology, podiatry, physiotherapy, occupational therapy and others in close collaboration with general practitioners (GPs).

Disease activity should be measured at each assessment using validated tools such as the Systemic Lupus Erythematosus Disease Activity Index 2000 (SLEDAI-2K) and the British Isles Lupus Assessment Group (BILAG) 2004, although these are not always used. For first-line therapy, antimalarials (e.g. hydroxychloroquine, chloroquine phosphate, mepacrine) are the cornerstone of treatment in SLE. All patients should be treated with antimalarials (unless contraindicated). In JSLE, in which disease onset is generally more aggressive, hydroxychloroquine and the early addition of conventional immunosuppressant is generally warranted. For all people with well-controlled SLE, minimal effective therapy is continued long-term. However, for those who either have intolerance or do not respond to first-line therapy with antimalarials, treatment will be escalated to various immunosuppressants, including biologic therapies (e.g. belimumab, rituximab), based on manifestations of disease, person-specific factors and funding criteria. Glucocorticoids may be used as a bridging therapy while a flare settles and/or a new immunosuppressant/biologic takes effect in the short term, with the aim to reduce or withdraw glucocorticoids once disease is controlled. However, many people with SLE continue unacceptable doses of glucocorticoids long-term. Indeed, results from a UK multicentre audit of the existing BSR guideline highlighted that 200/702 (28%) people with inactive SLE were on a maintenance dose of oral prednisolone [32].

In JSLE, although traditionally methotrexate, azathioprine and ciclosporin, along with cyclophosphamide for more severe disease, were also used, mycophenolate mofetil has generally become the initial immunosuppressant of choice [8]. These conventional immunosuppressants, including glucocorticoids, can be associated with side effects and toxicities. Therefore personalised and shared decision making is required in the choice of therapies that should take into account the predominant features of SLE and which therapies have the most suitable route of administration, frequency and routes of treatment, infection risk profile, complexity and risks for the individual. It is recognized that there can be other clinical challenges (e.g. attribution of cytopenias to lupus or cytotoxic drugs, difficulties in distinguishing manifestations of lupus disease activity from damage and comorbidities).

The NICE Technology Appraisal (TA752) recommended belimumab as an add-on treatment for active autoantibody-positive SLE in people with high disease activity despite standard therapy as defined by at least one serological biomarker (positive anti-dsDNA or low complement) and a Safety of Estrogens in Lupus Erythematosus National Assessment (SELENA)-SLEDAI score ≥10 [33]. Although rituximab is currently not licensed for treating SLE, it is available as a treatment option through the 2020 NHSE clinical commissioning policy for post-pubescent children or adults with moderate or severe refractory SLE with active disease who have failed to respond or have had adverse events to two or more immunosuppressive therapies (one of which must be either mycophenolate or cyclophosphamide, unless contraindicated). Disease activity is defined by at least one BILAG A and/or two B scores or a SLEDAI-2K score ≥6 points or requiring unacceptably high levels of oral glucocorticoids (e.g. >7.5 mg prednisolone in an adult per day), as well as having been assessed as not eligible for clinical trials or belimumab therapy [34]. Recent studies have demonstrated the efficacy and safety of new therapies including belimumab and voclosporin in LN [3, 4], anifrolumab in non-renal lupus [35] and obinutuzumab, a type 2 anti-CD20 monoclonal antibody, in patients with secondary non-response to rituximab [36]. These will be reviewed in the updated BSR guideline.

Despite the existing guidance, there is marked divergence in practice between centres, including management that is at times not consistent with the best evidence. A UK multicentre audit of the existing BSR guideline highlighted low documented compliance (<60% clinic visits) for audit standards relating to formal disease activity assessment, reduction of drug-related toxicity and protection against comorbidities and damage, particularly in general clinics compared with dedicated SLE clinics [32]. An updated guideline is needed on organization of clinical care within the UK healthcare system to harmonize care and define auditable standards.

### Who is the guideline for?

This guideline is intended for rheumatologists, dermatologists, nephrologists, immunologists, neurologists, respiratory physicians, obstetricians, GPs and other clinicians involved in the management of people with SLE; specialist nurses, pharmacists and allied healthcare professionals involved in their care; as well as people with SLE and their caregivers.

#### Equity considerations

Numerous gender, age, ancestral and sociodemographic factors are relevant in the management of SLE. The need for high-quality education, long-term community-based management and the use of specialized treatments means that language and cultural barriers to equitable and excellent care need to be considered and addressed.

### What will the guideline cover?

#### Who is the focus?

The guideline will address the care of people of all ages with SLE affected by any features or organ domains.

#### Settings

Settings that will be covered include primary, secondary and tertiary care within the UK.

#### Key areas that will be covered

We will search and update evidence in key areas when developing the guideline, namely diagnosis, assessment and monitoring; non-pharmacological intervention; pharmacological treatment and organizations or services for SLE within the NHS, including paediatric services and transition of young people to adult services. However, depending on the evidence available, it may not always be possible to provide guidance in all of the key areas; consensus agreement will be used in such cases.

#### Groups or areas that will not be covered

Neonatal lupus and those with lupus-like syndromes (e.g. monogenic interferonopathies). This guideline will not cover in detail the use of drugs in the management of pregnant females with SLE and will refer to the extensive review of drugs used in pregnancy and breastfeeding that has been recently published in a BSR guideline [37]. Specific management of complications/comorbidities associated with SLE and its treatment, including cardiovascular risk, osteoporosis, infection and cancer risk, will not be discussed in detail. These complications/comorbidities should be managed as for other patients with similar risk factors according to national and international guidelines. Thrombosis prevention and management will be limited to secondary aPL syndrome in people with SLE.

#### Related guidance

Related guidance includes the existing BSR guideline for adults with SLE [1], BSR guideline on prescribing drugs in pregnancy and breastfeeding: immunomodulatory anti-rheumatic drugs and corticosteroids [37], EULAR and EULAR–ERA-EDTA recommendations for adults with SLE (i.e. published in 2019, updated in 2023) [2, 38, 39], Kidney Disease: Improving Global Outcomes (KDIGO) Clinical Practice Guideline for the Management of Glomerular Diseases (published in 2021, being updated in 2023) [40], European recommendations for diagnosis and treatment of JSLE and for LN: the SHARE initiative (both published in 2017, with treatment section updated in 2022) [6–8], the 2021 British Association of Dermatologists guidelines for the management of people with cutaneous lupus erythematosus [41], the 2023 EULAR recommendations for the non-pharmacological management of SLE and systemic sclerosis [42], the 2020 Royal College of Ophthalmologists recommendations on monitoring for hydroxychloroquine and chloroquine retinopathy [43] and relevant current and future international lupus and LN guidelines and NICE and NHSE policies for SLE [33, 34, 44].

### Key issues and draft questions

While writing this BSR guideline scope, the working group identified many challenges facing clinicians and people with SLE warranting consideration and inclusion in these guidelines. We collated multiple questions and prioritized them during our meeting to make this task more manageable and in keeping with the scope of this guideline development process. The following key issues were selected and the associated draft questions related to them summarized. These key issues and draft questions will be used to develop more detailed review questions, which will guide the systematic review of the literature. The main outcomes that may be considered when searching for and assessing the evidence are clinical response, clinical flare, impact on health-related quality of life, organ damage accrual and daily glucocorticoid requirement, as well as associated morbidity, mortality, disability, educational attainment and employment.

#### Diagnosis of SLE

- What clinical and serological features should prompt consideration of a diagnosis of SLE in primary or secondary care?

#### Assessment and management

- What is the best way to assess people with SLE?
- What is the best way to monitor people with SLE?
- What is the role of a treatment target in the management of SLE?

#### Non-pharmacological intervention

- What non-pharmacological interventions are supported by evidence and/or expert consensus to inform a BSR guideline, including sun protection, smoking cessation, dietary advice, exercise, fatigue management strategies, measures to promote bone health and psychological support for people with SLE?

#### Pharmacological intervention

##### General approach

- What is the best evidence for the management of inactive or low disease activity SLE?
- What is the best evidence for monitoring of anti-malarial therapies?
- What is the best evidence for the management of moderate to severely active SLE?

##### Organ-specific approach

For the above evidence, we will highlight where evidence is specific to certain organ manifestations. In particular, we will review the following features in greater detail because recently there have been several specific trials for LN and cutaneous lupus, including topical therapies, which were not covered in the previous guideline:

- What is the best evidence for active LN induction treatment?
- What is the best evidence for LN maintenance treatment?
- What is the best evidence for management of cutaneous lupus erythematosus?

#### Organization of care

- How should care be delivered to optimize management of people with SLE?

### Timeline for publication of guideline

The guideline is expected to be published in 2024.

### Guideline working group

Edward M. Vital (Co-Chair, adult rheumatologist), Md Yuzaiful Md Yusof (Co-Chair, adult rheumatologist), Antonios Psarras (adult rheumatology trainee), Asad A. Zoma (adult rheumatologist), Arvind Kaul (adult rheumatologist), Bridget Griffiths (adult rheumatologist), Caroline Gordon (adult rheumatologist), Chris Wincup (adult rheumatologist), Christian D. Mallen (general practitioner), Christopher J. Edwards (adult rheumatologist), Clare Nash (paediatric pharmacist), Clarissa Pilkington (paediatric rheumatologist), Dalila Tremarias (adult patient representative), David Isenberg (adult rheumatologist), Elena Moraitis (paediatric rheumatologist), Eve M.D. Smith (paediatric rheumatologist), Hanna Lythgoe (paediatric rheumatology trainee), Heather Rostron (paediatric nurse specialist), Jade Skeates (physiotherapist), Kalveer Flora (adult pharmacist), Kate Armon (paediatric rheumatologist), Lindsey Cherry (podiatrist), Liz Lightsone (adult nephrologist), Michael W. Beresford (paediatric rheumatologist), Morgan Brown (adult patient representative), Naomi Maxwell (paediatric patient representative), Natasha Jordan (adolescent rheumatologist), Paul Howard (adult patient representative), Peter Lanyon (adult rheumatologist), Philip M. Laws (adult dermatologist), Rebecca Gilman (adult nurse specialist), Ruth J. Pepper (adult nephrologist), Sammy Ainsworth (paediatric patient representative), Sarah Skeoch (adult rheumatologist) and Stephen D. Marks (paediatric nephrologist).

## Data Availability

All data underlying this article are available in the manuscript.
